# Structure of the Bro1 Domain Protein BROX and Functional Analyses of the ALIX Bro1 Domain in HIV-1 Budding

**DOI:** 10.1371/journal.pone.0027466

**Published:** 2011-12-05

**Authors:** Qianting Zhai, Michael B. Landesman, Howard Robinson, Wesley I. Sundquist, Christopher P. Hill

**Affiliations:** 1 Department of Biochemistry, University of Utah School of Medicine, Salt Lake City, Utah, United States of America; 2 Department of Biology, Brookhaven National Laboratory, Upton, New York, United States of America; University of Hyderabad, India

## Abstract

**Background:**

Bro1 domains are elongated, banana-shaped domains that were first identified in the yeast ESCRT pathway protein, Bro1p. Humans express three Bro1 domain-containing proteins: ALIX, BROX, and HD-PTP, which function in association with the ESCRT pathway to help mediate intraluminal vesicle formation at multivesicular bodies, the abscission stage of cytokinesis, and/or enveloped virus budding. Human Bro1 domains share the ability to bind the CHMP4 subset of ESCRT-III proteins, associate with the HIV-1 NC^Gag^ protein, and stimulate the budding of viral Gag proteins. The curved Bro1 domain structure has also been proposed to mediate membrane bending. To date, crystal structures have only been available for the related Bro1 domains from the Bro1p and ALIX proteins, and structures of additional family members should therefore aid in the identification of key structural and functional elements.

**Methodology/Principal Findings:**

We report the crystal structure of the human BROX protein, which comprises a single Bro1 domain. The Bro1 domains from BROX, Bro1p and ALIX adopt similar overall structures and share two common exposed hydrophobic surfaces. Surface 1 is located on the concave face and forms the CHMP4 binding site, whereas Surface 2 is located at the narrow end of the domain. The structures differ in that only ALIX has an extended loop that projects away from the convex face to expose the hydrophobic Phe105 side chain at its tip. Functional studies demonstrated that mutations in Surface 1, Surface 2, or Phe105 all impair the ability of ALIX to stimulate HIV-1 budding.

**Conclusions/Significance:**

Our studies reveal similarities in the overall folds and hydrophobic protein interaction sites of different Bro1 domains, and show that a unique extended loop contributes to the ability of ALIX to function in HIV-1 budding.

## Introduction

Membrane remodeling is an essential component of many important biological processes, including cell division, membrane protein trafficking, and enveloped virus budding. Membrane deformations are typically controlled by peripheral membrane binding proteins that help to shape membrane and catalyze fission/fusion events (e.g., [1], [2], [3], [4]). One important cellular membrane remodeling system is the ESCRT pathway, which coordinates the “reverse topology” membrane deformation and fission events that accompany intralumenal vesicle formation at the multivesicular body (MVB), the abscission stage of cytokinesis, and the budding of many enveloped viruses (recently reviewed in [5], [6], [7]).

The human ESCRT pathway comprises more than 30 proteins that function in cargo sorting, membrane remodeling, and membrane fission. The recruitment of soluble ESCRT factors to different membranes begins when site-specific adaptor complexes bind directly to early-acting ESCRT factors such as ALIX (Bro1p in yeast) and/or the ESCRT-I complex. These factors help to concentrate protein cargoes, stabilize the necks of nascent vesicles, and recruit downstream factors. The late-acting ESCRT-III and VPS4 factors then act together to mediate membrane fission. The ESCRT-III subunits form helical (or spiraling) rings within the bud necks and recruit the VPS4 ATPase, which in turn uses the energy of ATP hydrolysis to remodel the ESCRT-III proteins, resulting in membrane fission and ESCRT factor release.

ALIX plays particularly important roles in abscission [8], [9] and in the budding of several different retroviruses, including EIAV and HIV-1 [10], [11], [12], [13]. ALIX is recruited to the midbody through direct interactions between the protein's C-terminal proline-rich region (PRR) and the adaptor, CEP55 [8], [9], [14]. Similarly, ALIX is recruited to sites of virus budding via interactions between the central ALIX V domain and YPX_n_L sequences (where X represents any residue and n = 1–3) within structural viral Gag proteins [10], [15], [16]. In both cases, the ALIX Bro1 domain then binds and recruits members of the CHMP4 subset of ESCRT-III proteins, thereby linking membrane-specific adaptors to the ESCRT-III/VPS4 fission machinery [9], [11], [17], [18]. These interactions may also regulate ESCRT-III stability and/or disassembly because interactions between Bro1p and Snf7p (the yeast homolog of CHMP4 proteins) can enhance the stability of ESCRT-III assemblies and inhibit their disassembly by Vps4p [19].

In addition to ALIX, the human proteome contains four other known Bro1 domain-containing proteins: HD-PTP (also called PTPN23) [20], BROX [21], RHPN1 [22] and RHPN2 [23], [24]. HD-PTP and ALIX share a similar domain organization (Bro1-V-PRR), except that HD-PTP also contains an additional C-terminal protein tyrosine phosphatase-like domain [25]. HD-PTP appears to function as the Bro1p homolog for ESCRT-dependent protein sorting in mammalian MVB pathways [26], [27]. The function of BROX is less clear, but this protein also likely functions in ESCRT-dependent membrane remodeling processes because BROX also binds CHMP4 [21] and, like other ESCRT proteins, becomes partially trapped on endosomal membranes upon overexpression of a dominant negative mutant of VPS4B [21]. BROX comprises a single Bro1 domain and has a C-terminal “CAAX” farnesylation site (where C is cysteine, A an aliphatic amino acid, and X any amino acid). The two human rhophilin proteins, RHPN1 and RHPN2, are Rho-GTP binding proteins involved in cytoskeletal dynamics [22], [24]. The full length RHPN2 protein reportedly does not bind CHMP4A [28], and neither protein has been clearly linked to the ESCRT pathway, although RHPN2 localizes to late endosomes and this localization is mediated by the protein's Bro1 domain [29].

In addition to connecting membrane-specific adaptors to the downstream ESCRT-III fission machinery, there are indications that Bro1 domains may make additional interactions that promote cargo sorting and membrane remodeling. For example, the Bro1 domains of ALIX, HD-PTP, BROX, and RHPN1 can bind the NC domains of HIV-1 Gag proteins and stimulate the release of virus-like particles [30], [31], [32]. Moreover, even BROX mutants that cannot bind CHMP4 retain some ability to stimulate virus budding, indicating that Bro1 domains can also act in other ways [31]. One possibility is that Bro1 domains may bind membranes and induce negative curvature. This model was first proposed because the crystal structure of the Bro1 domain of yeast Bro1p revealed a basic convex surface on the elongated, banana-shaped domain that could mediate membrane binding and thereby induce membrane curvature [33]. Although direct experimental evidence is lacking, analogous membrane bending (or sensing) activities have been well documented for BAR domains, which bind membranes using curved basic surfaces [34], [35]. Furthermore, the ability to promote negative membrane curvature could explain how highly divergent Bro1 domains can all promote the budding of minimal HIV-1 Gag constructs [31].

To date, high-resolution structural information has only been available for the Bro1 domains from yeast Bro1p [33] and the human protein ALIX [11], [36]. We reasoned that comparing additional Bro1 domain structures might help to identify key architectural features and we have therefore determined the crystal structure of human BROX. This new structure allowed us to identify conserved and unique elements present in the Bro1 domains of ALIX and BROX, and to test whether these structural elements contribute to ALIX stimulation of HIV budding.

## Results and Discussion

### BROX Expression and Crystallization

Human (His)_6_-BROX was expressed in *E. coli*, enriched by Ni^2+^ affinity chromatography, treated with TEV protease to remove the (His)_6_ tag, and then purified to homogeneity by anion exchange and gel filtration chromatography. The construct used in our studies spanned BROX residues 1–401, and therefore lacked 10 C-terminal residues (402–411), including the farnesylated Cys408 residue. The 47 kDa protein eluted from a gel filtration column as a single species with an apparent molecular weight of 57 kDa. This result indicated that recombinant BROX is a monomer because the protein has an elongated structure that will tend to increase its apparent molecular weight slightly (see below). In this regard, BROX apparently differs from ALIX, which can dimerize in an antiparallel orientation, with the V domains associating centrally and the Bro1 domains displayed at either end of the crescent-shaped complex [18].

BROX crystallized in space group C2 with three molecules in the asymmetric unit. The structure of the selenomethionylated protein was solved at 2.7 Å resolution using the MAD method, and the final structure was refined against a 2.5 Å resolution data set collected from crystals of the native protein (Table 1). The electron-density map was generally well defined, including for most side chains, except for a lack of density for the loop spanning residues 379–390, and marginal density for the terminal residues 391–401. The three BROX molecules in the asymmetric unit form a triangle in which the exposed hydrophobic Surface 1 from one molecule packs against the hydrophobic Surface 2 of a neighbor.

**Table 1:** Crystallographic Statistics for Brox(1–401).

Data Collection				
		SeMet		Native
Wavelength (Å)	0.97910	0.97940	0.96110	1.54178
Space group	C2	C2	C2	C2
Cell dimensions (Å,°)	a = 227.6	a = 227.7	a = 227.8	a = 228.0
	b = 67.2	b = 67.2	b = 67.3	b = 67.2
	c = 103.5	c = 103.6	c = 103.6	c = 103.6
	β = 96.6	β = 96.6	β = 96.6	β = 96.9
Resolution (Å)	40-2.7	40-2.7	40-2.7	40-2.5
	(2.8-2.7)	(2.8-2.7)	(2.8-2.7)	(2.59-2.50)
Completeness (%)	98.8 (98.5)	99.6 (97.2)	98.8 (98.9)	97.7 (96.5)
I/σ(I)	18.1 (3.6)	21.3 (3.0)	21.4 (3.3)	21.9 (2.6)
Rsym (%)	9.3 (45.3)	9.1 (47.2)	9.0 (49.0)	5.8 (48.4)
Number of unique reflections	43, 199	43, 233	43, 242	54, 427
**Refinement**				
Rfactor/Rfree (%)	19.8/24.8			
Number of protein atoms	9,269			
Number of water molecules	31			
Average B-factor (Å^2^)				
Protein atoms	60.5			
Water Molecules	44.0			
RMSD from ideal geometry				
Bonds (Å)	0.006			
Angles (°)	0.918			

Values in parentheses refer to the high resolution shell.

### BROX Structure

**Figure 1:**
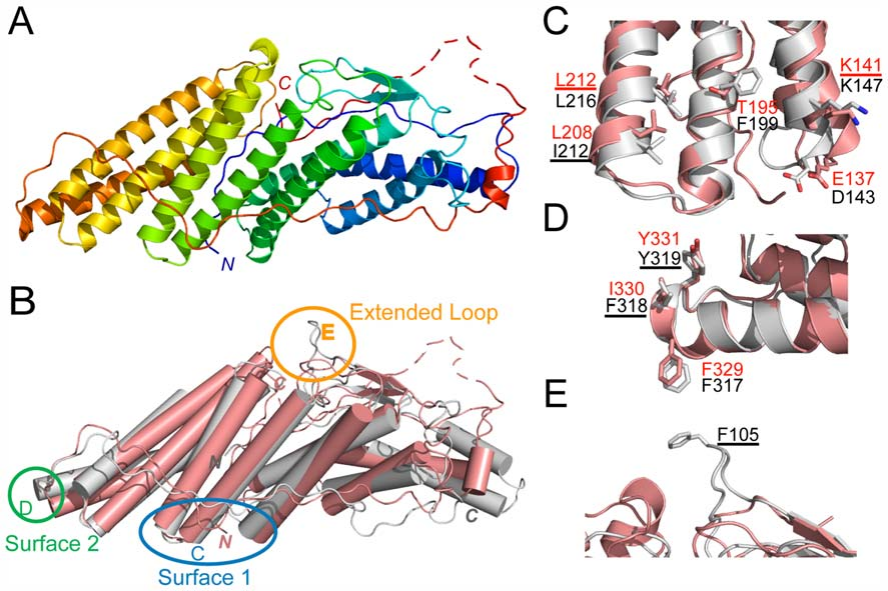
Structure of Human BROX and Comparison with the ALIX Bro1 Domain. (A) Rainbow ribbon diagram showing the structure of human BROX. The backbone is color coded from N-terminus (blue) to C-terminus (red). (B) Superposition of the structures of the Bro1 domains from BROX (salmon) and ALIX (grey, pdb 2XS1). Elements of interest are circled and shown as expanded views in (C–E).

BROX forms a single curved domain of overall dimensions: 90×37×38 Å. As expected, the structure resembles the Bro1 domains of ALIX and Bro1p [11], [33], with a core comprising three tetratricopeptide repeat (TPR)-like helical hairpins that pack in a right-handed solenoid (helices α4–9). The TPR core is flanked on the wider side by a two-stranded hairpin (β1, β2) and a helical bundle (α1–3). As in other Bro1 domains, an extended loop traverses the length of the domain on the concave surface (BROX residues 332–365). The C-terminus (red in Fig. 1A) forms a short helix (residues 366–372, drapes back across the domain (disordered residues 379–390), and then projects “upward” to terminate at the middle of the convex face (residues 391–401). Thus, the expected location of the Cys408 farnesylation site is consistent with the idea that the convex face binds membranes, although the final 10 BROX residues were not present in our structure and the penultimate residues may be mobile, as suggested by their limited crystallographic order.

### Comparison of the Bro1 domains from BROX, Bro1p, and ALIX

**Figure 2:**
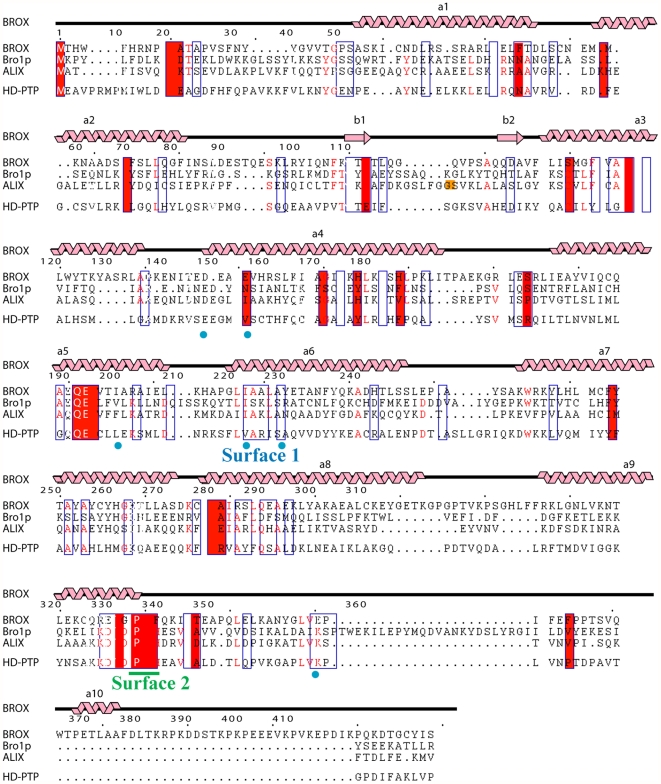
Secondary Structure of BROX and Structure-based Sequence Alignments of Different Bro1 Domains. BROX secondary structure is indicated above. Similar residues are boxed, identical residues are shown with a red background. Residues that contribute to Surfaces 1 and 2 are identified below in blue and green, respectively. ALIX Phe105 is shown on an orange background. Pairwise primary sequence identities and RMSD overlaps of the different Bro1 domains are BROX∶ALIX, 20%, 2.6 Å; BROX∶Bro1p, 20%, 3.1 Å; ALIX∶Bro1p, 24%, 2.8 Å. The Bro1 domains from the rhophilins RHPN1 and RHPN2 were not aligned here owing to their large primary sequence divergence.

**Figure 3:**
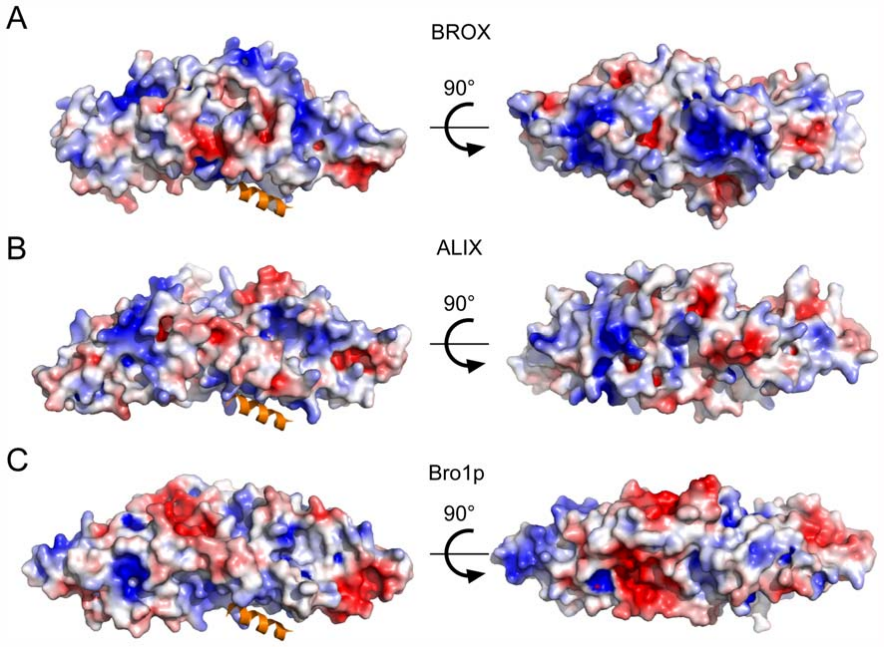
Electrostatic Surface Representations of the Bro1 Domains from BROX, ALIX and Bro1p. (A–C) Surface renderings of the Bro1 domains from the designated proteins, color-coded with basic regions in blue (kT/e = 4) and acidic regions in red (kT/e = −4). Two orthogonal views of each domain are shown. The C-terminal helix of CHMP4B (orange) is shown bound to the ALIX Bro1 domain in (B) (pdb 3C3Q), and for reference is shown modeled into analogous positions in the Bro1 domains in (A) and (C).

A structure-based sequence alignment of the Bro1 domains from Bro1p, ALIX and BROX is provided in Fig. 2. The primary sequence identities between the different Bro1 domains range between 20 and 24%, and the Cα atoms of the different known Bro1 domain structures align with RMSDs between 2.6 and 3.1 Å over the first ∼372 residues (Fig. 2, caption). All three Bro1 proteins contain two exposed hydrophobic surface patches that were first described in the structure of yeast Bro1p [33]. Surface 1 is located near the middle of the concave face (Figs. 1A and 1C), and corresponds to the CHMP4 binding site (Fig. 3A) [36]. Surface 2 is located at the narrow tip of the elongated domain (Figs. 1A and 1D). This surface appears to correspond to the site where Src kinases bind when ALIX residue Tyr319 is phosphorylated [37].

Despite their similar core structures, there are interesting differences between the Bro1 domains of BROX, Bro1p and ALIX: 1) only the ALIX Bro1 domain has an extended loop between β strands 1 and 2 that projects away from the convex face (Fig. 1E). 2) The BROX C-terminus is unique, presumably because the Bro1p and ALIX Bro1 domains are connected to downstream V domains, whereas BROX terminates at the _408_CYIS_411_ farnesylation site. 3) The detailed conformations of the helical bundles at the wide end of the domain differ in all three structures (Fig. 1B and [11]).

Hurley and colleagues have proposed that Bro1 domains bind membranes and generate negative curvature by wrapping the membrane along the basic convex surface [33]. As shown in Fig. 3A, the convex face of BROX does indeed have strongly basic character, as predicted by this model. The positions of the basic patches differ between the different Bro1 domains, however, because basic residues cluster near the center of BROX Bro1 domain, whereas they cluster at the narrow ends of the Bro1p and ALIX and Bro1 domains (compare Figs. 3A, 3B and 3C).

### BROX-CHMP4 Interactions

**Figure 4:**
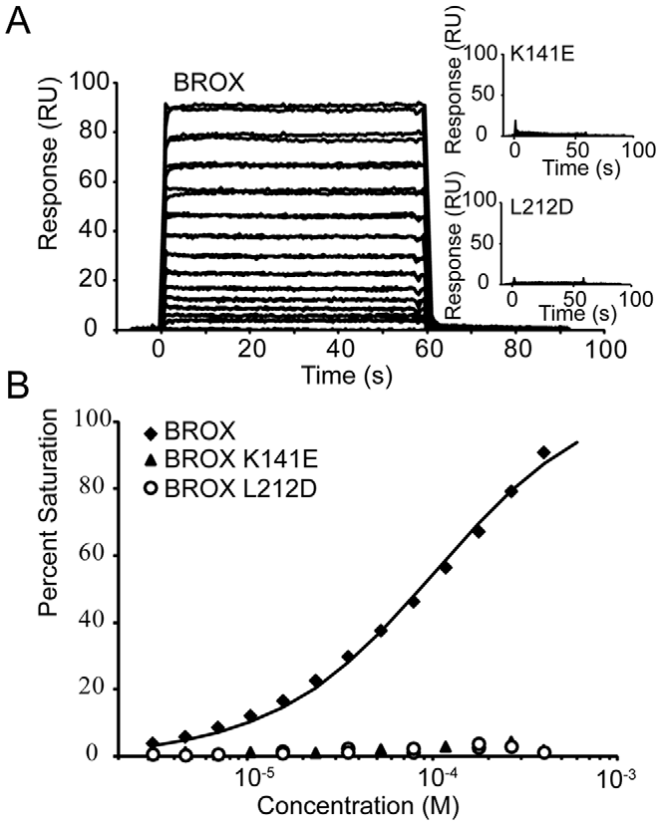
BROX Binding to CHMP4B205-224. (A) Biosensorgrams showing binding levels of different concentrations of wild type BROX (main graph) and BROX proteins (insets) with two different Surface 1 mutations (K141E and L212D) to immobilized GST-CHMP4B_205–224_. Binding studies were performed and analyzed as described in the Materials and Methods, and samples were measured in triplicate. (B) Biosensor isotherms for wild type BROX, BROX_K141E_, and BROX_L212D_ proteins binding to immobilized GST-CHMP4B_205–224_.

Previous biochemical and structural studies have revealed that C-terminal helices from the CHMP4 family of ESCRT-III proteins bind across Surface 1 of the ALIX Bro1 domain (Fig. 3) [36]. Immunoprecipitation and mutagenesis experiments indicate that CHMP4 proteins also bind the Surface 1 sites on BROX and HD-PTP [21], [26], [31]. These interactions have not been reconstituted with pure proteins, however, and we therefore performed biosensor experiments to quantify the binding of recombinant human BROX to an immobilized C-terminal fragment from CHMP4B (GST-CHMP4B_205–224_). As shown in Fig. 4, BROX bound to GST-CHMP4B_205–224_, but not to a control GST surface. The dissociation constant for the BROX-CHMP4B_205–224_ interaction was 98±22 µM, which is similar to that of the ALIX Bro1-CHMP4B_205–224_ interaction (48±6 µM) [36]. Two different single point mutations within BROX Surface 1 (K144E or L212D) abolished the interaction with CHMP4B entirely (Fig. 4), confirming that CHMP4B binds to the same site in ALIX and BROX. As shown in Figs. 1C and 2, the CHMP4 binding residues within Surface 1 are generally conserved (or conservatively substituted) between ALIX, BROX and HD-PTP, and it is therefore likely that all of these Bro1 domains bind the C-terminal tails of the CHMP4 proteins similarly.

### Functional Importance of ALIX Bro1 Elements in Stimulating HIV-1 Budding

**Figure 5:**
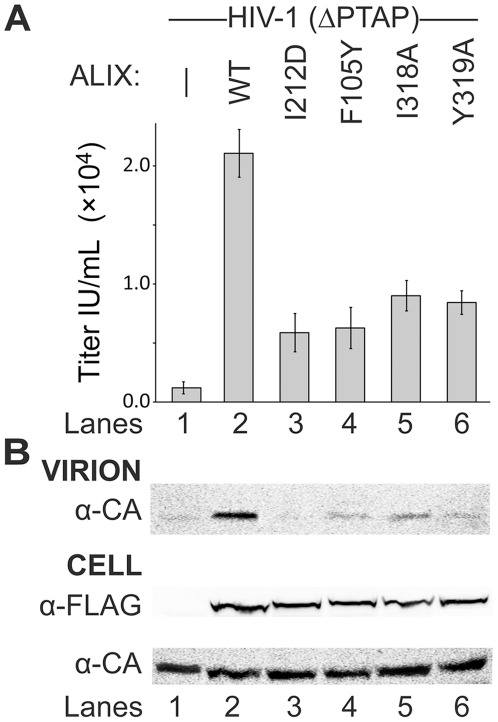
Functional Analyses of ALIX Bro1 Elements in HIV-1 Budding. Levels of HIV-1 ΔPTAP infectivity and release upon co-expression with: an empty vector (lane 1, negative control), a vector expressing wild-type ALIX (lane 2, positive control), and with ALIX Bro1 domain mutants (lanes 3–6). (A) Infectious titers were analyzed using flow-cytometry to determine the percentage of GFP-positive cells. Error bars in infectivity assays show standard deviations from five separate infectivity experiments. (B) Western blots showing levels of virion-associated CA protein release (top panel), and cellular levels of exogenous ALIX and viral Gag proteins (lower two panels).

We next tested whether the different Bro1 elements identified in our structural studies contribute to the ability of ALIX to stimulate virus release. Our experiments took advantage of the fact that mutant HIV-1 constructs that lack the _11_PTAP_14_ late domain sequence in the p6^Gag^ protein cannot recruit the ESCRT-I complex normally, and are therefore only released efficiently when functional ALIX proteins are overexpressed [11], [17]. As shown in Fig. 5, overexpression of wild type ALIX strongly stimulated the release of a HIV-1_NL4-3_ (ΔPTAP) vector, as assayed by the increased levels of virion-associated HIV-1 CA proteins released into the culture supernatant (Fig. 5B, Panel 1, compare lanes 1 and 2) and by increased vector titers (18-fold above the control, Fig. 5A). In contrast, a control ALIX construct with a mutation in the CHMP4 binding site within the Bro1 domain (ALIX_I212D_) stimulated virion release and infectivity much less (Fig. 5B, Panel 1, compare lanes 2 and 3, and note the 3.6-fold titer reduction vs. wild type ALIX in Fig. 5A). Thus, as has been shown previously [11], [17], CHMP4 binding by the ALIX Bro1 Surface 1 contributes to the protein's ability to function in virus budding. Similarly, two different mutations in hydrophobic Surface 2 residues of ALIX (I318A and Y319A) each reduced the stimulation of virion release and infectivity by 2–3-fold, in good agreement with a previous report [17]. In all cases, cellular CA levels were not altered by overexpression of the ALIX constructs (Fig. 5B, Panel 3) and the ALIX proteins were expressed at similar levels (Panel 3).

As illustrated in Figs. 1B and 1E, the most unique feature of the ALIX Bro1 domain is the extended surface loop that projects away from the convex face. Residue Phe105 resides at the very tip of this loop, suggesting the possibility that this exposed hydrophobic residue might perform an important function. We therefore performed analogous experiments to test the functional requirement for the exposed Phe105 residue in ALIX stimulation of HIV-1 budding. As shown in Fig. 5, an ALIX protein with a conservative Tyr mutation at residue 105 (ALIX_F105Y_) stimulated HIV-1 virion release and infectivity less effectively than the wild type ALIX protein (lanes 4, 3.4-fold titer reduction), even though the mutant and wild type proteins were expressed at similar levels (Fig. 5B, Panel 2, compare lanes 2 and 4). Thus, the F105Y mutation impairs the ability of ALIX to function in virus budding. Other control experiments (not shown) also behaved as expected in that overexpression of the different ALIX constructs had smaller effects on: 1) the release and infectivity of HIV-1 vector constructs that carried mutations in *both* the PTAP and YPX_n_L late assembly domains (because these constructs lacked the primary ALIX binding site within p6^Gag^, and therefore responded poorly even to the wild type ALIX protein), and 2) wild type HIV-1 vectors (because these constructs were already released well owing to their ability to recruit ESCRT-I, although we did reproducibly see some degree of unexplained stimulation for the ALIX_Y319A_ mutant).

Together with previous work, our studies demonstrate that at least three different structural elements within the Bro1 domain of ALIX contribute to the protein's ability to function in HIV-1 release. Specifically, we have confirmed the functional importance of Surfaces 1 [11], [17] and 2 [17], and demonstrated the functional importance of the Phe105 residue at the end of the extended beta hairpin loop. While this manuscript was under review, a related study appeared online [38]. Our work is in excellent agreement because both studies present similar crystal structures of the human BROX protein, and both use mutational analyses to demonstrate that the extended Phe105 loop is required for ALIX functions in virus budding.

The functions and/or binding partners of Surface 2 and the extended Phe105 loop are not yet known with certainty. Surface 2 has been implicated in binding Src kinases [37]. However, we believe that Src is unlikely to be the key positive-acting binding partner for Patch 2 in ALIX-mediated virus budding because: 1) The phosphorylatable Tyr319 phenol oxygen can be removed without loss of ALIX-mediated virus budding activity [11], 2) Unlike the more detrimental Y319A mutation, the more conservative Y319F mutation retains virus budding activity [11] yet inhibits Src binding [37], implying that Src binding is not required for budding activity, and 3) Loss of Src binding is expected to *activate* (rather than inactivate) ALIX because Src binding and phosphorylation of Tyr319 reportedly antagonize ALIX activity [37]. Interestingly, none of the single point mutations that we tested abolished ALIX activity entirely (Fig. 5, compare lane 1 to lanes 3–6). These data therefore provide further support for the idea that Bro1 domains perform multiple different functions in ESCRT-mediated membrane remodeling processes.

## Materials and Methods

### BROX protein expression and purification

BROX coding sequence was amplified from the human open reading frames (ORFs) from the Mammalian Genome Collection [39] and cloned into pET151/D TOPO vector (Invitrogen). Quickchange mutagenesis (Stratagene) was used to create expression constructs for pET151D/TOPO-BROX(1-401) (WISP11-298), pET151D/TOPO-BROX-K141E (WISP11-299), pET151D/TOPO-BROX-L212D (WISP11-300) based on pET151D/TOPO-BROX (WISP11-297). The proteins were expressed with N-terminal 6×His affinity tags in BL21(DE3) Codon+ (RIL) *E. Coli* in 2 L of ZYP-5052 auto-induction media [40]. Cultures were grown in baffled flasks at 37°C for 6–8 hours with vigorous shaking, and then shifted to 19°C and grown to saturation over 16–18 hours. SeMet BROX(1–401) was prepared by expression in PASM-5052 cells [40]. Cells were resuspended and treated with lysozyme (2.5 mg/ml in lysis buffer: 50 mM Tris pH 8.0, 300 mM NaCl, 5% glycerol, 10 mM imidazole) and sonication, and the lysate clarified by centrifugation. The protein was bound to a Ni^2+^ matrix, washed with 20 column volumes of lysis buffer, and eluted with 50 mM Tris pH 8.0, 100 mM NaCl, 5% glycerol, and 250 mM imidazole. Fractions containing the protein were pooled and treated with 0.2 mg TEV protease while being dialyzed against 2 L 25 mM Tris pH 8.0, 100 mM NaCl, 1 mM DTT, and 5% glycerol (18 h, 23°C). The cleaved protein was collected as flow-through from a second Ni^2+^ resin column and applied to a HiTrap Q Sepharase Fast Flow Column (GE Healthcare), and eluted by a NaCl gradient from 25 mM to 1 M (25 mM Tris pH 8.8, 1 mM DTT, and 5% glycerol). Finally, the protein was purified to homogeneity by size exclusion chromatography (Superdex 75, GE Healthcare, 10 mM Tris pH 8.0, 100 mM NaCl, 1 mM DTT). This procedure typically yielded 20 mg of pure monomeric native BROX.

### BROX crystallization and structure determination

Crystals of wild type and SeMet BROX (10 mg/ml) were grown at 13°C in sitting drops (reservoir: 20% PEG 1500, and 0.1 M MMT pH 6.6) and cryoprotected in a solution of reservoir components made up with 30% glycerol. Diffraction data were collected from SeMet protein crystals to 2.5 Å resolution at three wavelengths at beam line X-29 of the National Synchrotron Light Source and processed using HKL2000 [41]. The BROX structure was determined by the MAD method using the PHENIX AutoSol wizard [42], [43], [44], [45], [46]. Fourteen Se sites were identified from the MAD data, and the ALIX Bro1 domain structure was used to guide model building. The model was refined against 2.5 Å resolution native data, collected on a rotating anode source, in REFMAC5 with 3-fold NCS in the CCP4 suite [47] and in PHENIX with TLS refinement [42], [48], [49] to R/R_free_ values of 19.2/25.5 (Table 1).

### Biosensor binding studies

Binding experiments were performed using a Biacore 2000 instrument. CM5 sensor chips were derivatized with anti-GST antibody using amine coupling and were used to capture GST-CHMP4B_205–224_ [36] or GST alone from crude *E. coli* expression lysates. Pure BROX proteins were injected in triplicate in a 1.5-fold dilution series, starting at 400 µM. All interactions reached equilibrium rapidly and dissociated within seconds during the dissociation phase. Equilibrium binding responses were fit to 1∶1 binding isotherms to obtain equilibrium constants.

### Assays for Bro domain function in HIV-1 ΔPTAP release

HIV-1 virions were produced using a vector system and analyzed using methods similar to those described previously [16]. Virions were produced in 293T cells (4×10^5^ cells/well in 6-well plates) following calcium-phosphate (Clontech) co-transfection of the following plasmids: 1.35 µg of an HIV-1 RΔ8.2 construct (either wild-type (WISP04-151) or ΔPTAP (WISP04-150)), 1.35 µg pLOX-GFP [50], 0.4 µg pHCMV-VSV-G (WISP98-16) and 1 µg of the control construct (pCI-FLAG-EV(WISP02-31), or the expression constructs for ALIX (WISP03-308), ALIX_I212D_ (WISP11-401), ALIX_F105Y_ (WISP11-402), ALIX_I318A_ (WISP11-403), or ALIX_Y319A_ (WISP11-404)). The medium (2 ml/plate) was replaced 8 hr after transfection, and the supernatant was harvested 16 hr later and syringe-filtered through 0.45 µm membranes. Cells were harvested with cold PBS and lysed in 200 µL of RIPA (50 mM Tris, 150 mM NaCl, 0.1% SDS, 0.5% Sodium Deoxycholate, 1% NP40 and complete protease inhibitor cocktail (Sigma)). The supernatant was collected and adjusted 1∶3 with 3X SDS loading buffer. Virions were collected by pelleting 1 mL of medium through a 250 µL 20% sucrose cushion. The supernatant was removed and the virion pellet was dissolved in 60 µL of 2X SDS. Western blots were performed on the cell and virion samples using anti-CA, 1∶3000 (NIH AIDS Research & Reference Reagent Program, Catalog #3537) and anti-FLAG, 1∶3000 (Sigma, Catalog #F7425) and analyzed using an Odyssey imaging system (Li-COR, Inc.). Vector titers were determined using serial dilutions of vector preparations added to 293T target cells (2×10^5^ cells/well in 6-well plates) for 12 h at 37°C. The medium was replaced with normal culture medium for 60 h at 37°C, and the transduction efficiency, determined as the percentage of green fluorescent protein (GFP)-positive cells, was measured using flow-cytometry.
